# Patient perceptions and experiences of medication review: qualitative study in general practice

**DOI:** 10.1186/s12875-022-01903-8

**Published:** 2022-11-22

**Authors:** Deborah McCahon, Polly Duncan, Rupert Payne, Jeremy Horwood

**Affiliations:** Centre for Academic Primary Care, Population Health Sciences, Bristol Medical School, University of Bristol, Bristol, BS8 2PS UK

**Keywords:** Clinical medication review, Patient experience, Medicines optimisation, Primary care, Clinical pharmacist

## Abstract

**Background:**

Clinical medication reviews are a recognised strategy to address polypharmacy, a key part of general practice and positively associated with patient safety and clinical effectiveness. To date there has been little investigation of the patient perspective of medication reviews.

**Objective:**

To explore patient experiences of medication review including the processes and activities that led up to and shaped the review.

**Methods:**

Qualitative interview study within 10 general practices in Bristol. Participants were adults with polypharmacy (≥ 4 medications) and ≥ 2 long-term conditions who had a record of medication review with either a GP or pharmacist. Interviews were transcribed verbatim and analysed thematically using a data driven approach. Co-design work was undertaken with four patient and public involvement advisers to design and develop resources to support patient preparation for medication review.

**Results:**

Twenty-one patients were interviewed (10 female, mean age 73 years, range 59–88 years). Medication review was viewed as an opportunity to assess the effectiveness and need for medications. Participants expected the review to focus upon medication related concerns, side-effects and symptoms. Those who were newer to review, were uncertain of the intended purpose, and described their review as a box-ticking exercise. Some participants were unfamiliar with the role of the pharmacist and expressed a lack of confidence in their clinical skills and knowledge. Face-to-face consultation and relationship continuity were considered important for efficient and effective medication review. Results informed co-production of a patient information leaflet to facilitate greater patient engagement and involvement in medication review.

**Conclusions:**

A lack of understanding of the rationale for medication review can limit the value patients attach to these healthcare encounters. Improved prior communication and information around the intended purpose and potential benefits of medication review may enhance patient engagement and improve patient experience and outcomes.

**Supplementary Information:**

The online version contains supplementary material available at 10.1186/s12875-022-01903-8.

## Background

Medicines optimisation is critical for addressing the growing issue of polypharmacy in the UK (the use of multiple medicines by individual patients) and has been promoted by independent organisations and official national guidelines [1–4]. Medicines optimisation emphasises patient engagement and involvement in decision making. In addition to promoting the evidence-based use of medicines, it encourages prescribers to focus upon patient related experiences and goals with a view to improving adherence, safety and effectiveness of medication. Regular medication review is recognised as a key priority for implementation and mechanism for making medicines optimisation part of routine practice in the UK [4].

NICE define medication review as a “structured, critical examination of a patient’s medicines with the objective of reaching an agreement with the patient about treatment, optimising the impact of medicines, minimising the number of medication related problems and reducing waste” [4]. A number of different forms of medication review are carried out in UK primary care, including prescription reviews, comprising a technical review of the patient’s prescriptions, and clinical medication review, usually conducted with the patient present and considering clinical issues such as effectiveness or adverse effects. For patients with long-term conditions, the Quality and Outcomes Framework (QOF) incentivises annual disease-specific review as a means of disease-specific treatment optimisation [5]. The focus of such reviews is inherently disease-specific however a more general review of medicines used to treat coexisting conditions can take place during this consultation (i.e. simultaneous disease-specific treatment review and clinical medication review). Where need is established, a disease specific-treatment review may also trigger a subsequent clinical medication review. In UK general practice, management of long-term conditions, mainly hypertension, diabetes, and respiratory disease, is often within the pharmacists’ scope of practice [6].

Different approaches to clinical medication review have been implemented across the world. In the Netherlands, pharmacists in different settings conduct clinical medication reviews with elderly patients with polypharmacy to increase the effectiveness of medication therapy as well as contribute to deprescribing [7]. This is usually performed based upon the STRIP method (Systematic Tool to Reduce Inappropriate Prescribing) [8]. In Australia, general practitioners refer patients to a pharmacist for a review of their medicines management needs, to optimise therapy and prevent medication related harm. As part of the review, the pharmacist consults with other allied healthcare professionals and suggests changes to therapy to the general practitioner [7]. Similarly in Slovenia, clinical pharmacists in healthcare centres undertake clinical medication reviews using several sources of information including patient interview, medication histories and clinical data and provide recommendations for change to the patient’s general practitioner [7]. Both prescription review and clinical medication reviews have traditionally been conducted by doctors in UK general practice because of their access to the full medical record and role in the coordination of care. This role is now increasingly the responsibility of practice pharmacists. Not all general practices in the UK have an affiliated pharmacist, although there are considerable regional differences, and numbers are generally growing with a national programme to encourage the use of clinical pharmacists in the general practice setting [9, 10]. These pharmacists are employed by the practice and carry out non-dispensing roles, including medicines reconciliation, reviewing prescription requests, and clinical patient-facing activities. Structured clinical medication review and medicines optimisation are now a service requirement for Primary Care Networks (PCN; clusters of practices covering around 40,000 patients delivering coordinated, integrated community-based health services) across England, with clinical pharmacists attached to PCNs having key roles in delivering patient-facing medicines optimisation services [11].

The nature and quality of clinical medication review in current routine clinical practice is questionable. Evidence suggests that such reviews have largely been uncoordinated, unfocussed and unstructured GP-led activities which rarely result in changes in prescribing because GPs prioritise efficiency over quality [10]. Full patient involvement from the outset is desirable to maximise patient benefit and meet the aims and intentions of medicines optimisation policies [12], although most studies suggest the patient role in review is limited to information sharing [13]. Little is known about patient expectations, their experiences of person-centred care, or how they prepare for an active role in medication review in general practice. Since patient experience has been positively associated with clinical effectiveness and patient safety [14], the aim of this study was to gain a better understanding of patient perceptions and experiences of medication review as undertaken in routine general practice, including the processes and activities that led up to and shaped the review.

## Methods

### Interview sampling and recruitment

Potential participants were sampled from 10 general practices across the Bristol, North Somerset and South Gloucestershire Clinical Commissioning Group (CCG) area (where around two-thirds of practices employ a pharmacist). Practices were purposively selected in relation to patient list sizes and high and low social-economic deprivation areas. Participating practices searched their electronic records to identify community dwelling adults (aged ≥ 18 years), receiving ≥ 4 repeat prescription medications for a minimum of two long-term conditions who had received a clinical medication review with a GP or a pharmacist in general practice, during a face-to-face or telephone consultation within the preceding 12 weeks. Medication reviews that were instigated by the patient or invited by the practice were included. Individuals unable to provide written informed consent were excluded. Invitation letters were sent by each practice to the 15 eligible individuals who had mostly recently received a medication review (total number of invitations sent = 150). The expected positive response rate to the invitation mailout was 25%, providing a sample of 37 potential participants. To ensure maximum variation amongst interviewees, patients were selected for interview in relation to their age, gender, number of repeat medicines and number of chronic conditions. Sampling of participants continued until no new insights or themes were being captured during the interviews. To encourage participation by those patients who may have been unaware that their medication had been reviewed during a recent routine consultation, the invitation letter explained that the study aimed to capture a range of experiences and views and included the date of the patient’s most recent medication review.

### Interview procedures

In depth interviews took place between May and September 2018. All interviews were conducted by DM (female, PhD, experienced primary healthcare researcher) either via telephone or face-to-face at the individuals’ homes. Before interview, written (for face-to-face interviews) or verbal recorded consent (for telephone interviews) was gained. DM had no prior relationship with study participants. Interviews were conducted between DM and the participant with no one else present. Interviews took between 13–46 min (mean 30 min). Participants were asked to self-report; number of chronic conditions and regular medicines on repeat prescription, whether they recalled the medication review appointment referred to in their study invitation letter, how the review was instigated (e.g.via invitation from the practice or requested from the patient), the profession of their reviewer and the mode of consultation (in-person or via telephone). To ensure that each interview covered a range of core topics, a topic guide was developed in collaboration with a Patient and Public Involvement (PPI) advisor. Additional questions were asked to follow-up on topics requiring further explanation and enable discussion of subjects salient to the participant. The topic guide was amended to ensure that issues emerging from the analysis were explored in future interviews. This approach was used to avoid, as far as possible, imposing the interviewer’s framework of meanings onto the patient accounts.

### Data analysis

Interviews were audio recorded, transcribed verbatim, anonymised, checked for accuracy and imported into software for qualitative data analysis (NVivo, version 11) to aid indexing and management of data. Data analysis began shortly after data collection started and was ongoing and iterative.

Thematic analysis [15], utilising a data-driven inductive approach [16], was used to scrutinise the data to identify patterns and themes of salience for participants and across the dataset. DM and PD (academic GP) conducted initial line-by-line coding to construct a draft coding frame. A subset of interviews were independently double coded by DM, PD and JH to achieve coding consensus and ensure rigour. Key themes were discussed with JH (psychologist) and RP (GP and clinical pharmacologist) to ensure credibility and external validity considering alternative explanations and diverse cases.

### Co-design phase

Four Patient and Public Involvement (PPI) advisers were invited to co-design an output to facilitate utilisation of research findings by patients and healthcare professionals. Findings were presented to advisors alongside suggestions for outputs including a medication review information leaflet or an infographic for display within general practices. Advisers were asked to review the research findings and comment upon the suitability and potential utility of the suggested outputs and propose alternatives. A teleconference meeting with the advisers was convened to agree the output for production and discuss the content and layout. Based upon decisions made during this meeting, researcher DM produced a first draft which was circulated via email for comments. DM amended the document based upon feedback received and recirculated. A second teleconference was convened to agree and finalise the document.

## Results

Interviews were conducted with 21 participants; 10 females and 11 males with a mean age of 73 years (range 59–88 years) (see Table 1). The median number of self-reported repeat prescription medicines and chronic conditions was 4 (range 3–10) and 3 (range 2–3) respectively. Eleven of the 21 participants interviewed recalled their review, 5/11 had contacted their practice to request a review, 10/11 reported having a face-to-face review consultation and nine/11 stated that their reviewer was a GP. Ten of the 21 participants interviewed could not recall a recent first-hand experience of medication review and provided perceptions and views on medication review in general.

**Table 1:** Characteristics of the 21 participants interviewed

	**N (%)**
**Sex**	
Male	11 (52)
**Age Group (years)**	
< 60	1 (5)
60–69	7 (33)
70–79	6 (29)
80–89	7 (33)
**Median Number of self-reported repeat medicines (range)**	4 (3–10)
**Median Number of self-reported chronic conditions (range)**	3 (2–5)
**Participants unaware of or unable to recall the medication review**	10 (48)
**Participants aware of or able to recall the medication review**	11 (52)
Patient contacted the practice to arrange review	5 (45)
Practice invited the patient to attend review	6 (55)
Face to face consultation	10 (91)
Telephone consultation	1 (9)
GP-led review	9 (82)
Pharmacist-led review	2 (18)

Analysis led to the identification of four over-arching themes: patient understanding of the purpose of medication review; motivation to attend; perceptions of personal necessity and benefit; and preferences for the organisation and delivery of medication review. The themes are described below with the use of verbatim quotes. The participants’ awareness of a recent clinical medication review is presented alongside each quotation (e.g. ‘aware’ is used alongside quotes from participants who recalled their review and ‘unaware’ is used to indicate quotes from participants who were not aware they had received a review).

### Understanding of the purpose of medication review

Knowledge of medication review was derived from direct experience or sight of a review “due date” listed on prescription paperwork. Some reported ignoring the review “due date” on their prescription paperwork as they expected the practice to proactively contact them if a review was necessary.

it says prescription treatment to patient review date … they would like to review it but nobody’s ever come and asked me about it (Mr Q, unaware)

Participants who recalled having only one previous clinical medication review described feeling uninformed and uncertain of the intended purpose of a medication review so developed their own rationale for it. Several assumed that the aim of a review was to evaluate treatment effectiveness and identify problems with medicines. Others saw the review as part of the repeat prescribing process and necessary for continuation of medicines.

*I didn’t know if the intention was to take me off or … to see … if I needed them still. I wasn’t quite sure what it was going to involve or how long or in what depth (Mrs C, aware)*

One participant voiced concerns that the intended purpose might be to stop medicines, and another thought the review was needed to evaluate the quality of care being provided within a nurse-led chronic disease clinic.

*I didn’t know *whether* it was gonna be something …totally different to what I’d already experienced … with the nurse … I thought … they were gonna … say ‘You’ve done this for so long, we’ve got to move you onto this because … I don’t know … whether they were checking up on what the nurse was doing. (Mr G, aware)*

During medication review, participants expected their reviewer to ask about side effects, symptoms, and problems with the use of each medicine. Some hoped their reviewer would provide detailed information about their medicines, indications, and possible drug-drug interactions.

*Are there any adverse side effects? *Do* I feel happy with the way things are going? Do I want to change anything … just really sort of going over how I feel about taking the meds and whether they’re working (Mr I, aware)*

*I didn’t go with any *expectations* of anything new happening and I just wanted to know what was going on with the medicines and which ones I was taking for what… I just needed confirmation of what I was taking was right for what was wrong (Mrs H, aware)*

Changes to existing medicines during medication review were generally not anticipated by those more familiar with such reviews. One participant felt that reviewers were reluctant to make changes to medicines, particularly where the patient reported that their medication was not problematic, and symptoms were well controlled.

*you just think what a *nuisance* to go and … they say, “You’re still on them. Yes, everything’s all right, okay.” They don’t want to upset the status quo, which is sensible (Mrs O, aware)*

### Motivation to attend

Participants described being motivated to attend medication review as the appointment was timely, coinciding with medicines-related concerns or questions.

*I thought I shouldn’t be on so *and* so anymore and I thought there was one or two I needed to discuss so I went in (Mrs T, aware)*

Those who had little contact with their practice, described wanting to attend because medication review offered an opportunity to reassess the need for their medicines and the possibility to reduce the number of medications they were taking.

*If they find that things have settled *and* I can come off of one of them all the better … unless I have a problem then I don’t get to talk to anybody … and they don’t do any tests to see whether I am improving or I’m just staying still (Mr F, aware)*

Those receiving prescriptions from several different clinics or doctors were pleased to be offered an opportunity for an evaluation of their medicines together within the wider clinical context.

*I thought then that it was a good idea … because otherwise it’s just add on this one, take away that one, without looking at the whole picture (Mrs U, aware)*

### Perceptions of benefit and necessity

Medication review was considered beneficial by some because it provided assurance around medication effectiveness and made them feel looked after. Those who were new to medication review, described their experience as brief and uneventful and likened it to a box ticking exercise. Some participants described their reviewer as unprepared, apathetic, and questioned whether their medication review appointment was an appropriate use of resource. Participants also talked about medication review being beneficial for others, particularly those who lacked understanding of why they were taking or how to take their medicines.

*nothing much happens. It’s just, “How are you, all right?” “Yeah, okay, fine.” that’s really it so it’s a box ticking exercise ... I don’t think it is a good use of time because I don’t feel they’re [GPs] really into it... for patients like myself …it’s just hassle … some people don’t know their medicines, don’t know what they are, don’t know what they do and they’re just taking them… or don’t take them … so they do need a review (Mr N, aware)*

Medication review was considered unnecessary by those who were receiving fewer prescription medicines as they perceived their medication regimen as uncomplicated or low risk. These participants tended to feel competent in self-managing their medicines and to consult their GP with medication-related problems as they arose.

*In my case I don’t think *it’s* necessary because, … it’s … relatively straightforward medications. There’s no reason to change them unless the underlying reason is getting worse or getting better … it seems a bit pointless to waste the doctor’s time … when there’s no problem ... I take quite an interest in these sorts of things and [am] prepared to take action if it’s necessary. (Mr M, unaware)*

Others considered medication review to be unnecessary because they were already receiving holistic care and medicines were often discussed within their routine consultations with their GP.

*when I go in to see them [GPs] they do tend to look at all the things I’m on … I get the feeling that I’m being looked at in the whole rather than just for the specific (Mr K, unaware)*

### Preferences for the organisation and delivery of medication review

Some participants stated that their experience would have been improved if they had a better understanding of the aim of medication review. Additional prior communication in this regard was thought helpful for avoiding misconceptions and anxieties related to withdrawal of medicines. A prompt to prepare for the review through reflection upon the use of and need for medicines was also thought to be beneficial. Others argued that prior preparation was unfeasible in the absence of any medication related problems or unnecessary because the reviewer has access to the patients’ full medical record during the review.

*I think the anxiety would be either *they* [the patient] are reliant on them [medication], … and there’s a chance I might get taken off it …Or they’re gonna be involved in a discussion, which they don’t feel able to have proper input into around the clinical decision (Mr D, unaware)*

*if there was a letter saying we *noticed* that you’re due for a review … this would be a good chance for you to ask us any questions and for us to make sure that you’re receiving the right medication and ones that are actually necessary … have a think before…and that’s what we’ll discuss, that would be quite useful (Mrs C, aware)*

Participants conveyed a preference for a reviewer whom they were familiar with and who could provide continuity of care. This was important because it made the review more time efficient and negated the need to discuss medical and drug histories.

*Because you know the GP, *you’ve* known them for the thing the tablets is for… there’s a lot of history behind things, whereas the pharmacist wouldn’t have that… (Mrs U, aware)*

Several participants recognised pharmacists as specialists in medicines and this, alongside longer appointment times than for GPs, was considered advantageous. Those who had first-hand experience of a pharmacist-led review regarded pharmacists as having greater knowledge of medicines than GPs.

*A lot of things that were sort of *happening* and I didn’t understand why and it’s difficult to talk to the doctor because they have limited time … so talking to the pharmacist I must admit it set my mind at rest (Mrs H, aware)*

*My experience was the pharmacist, had all the information that the doctor would have* *… they appear to interpret the readings the same … if there is a problem … tablet needs to be changed … I would have thought the pharmacist might know off the top of her head … [laugh], a little bit quicker than the doctor would (Mr F, aware)*

A few participants expressed a lack of confidence in the clinical knowledge and skills of pharmacy professionals. Several participants were unaware that pharmacists had roles in general practice. Participant’s opinions of pharmacists were formed based on their experiences of discussing medicines with pharmacists in a community setting when medicines were being dispensed or during a Medication Use Review (e.g. an NHS-funded community pharmacy-based service for review of the patient’s medicines taking behaviour, knowledge and ability, conducted without access to medical records). These participants questioned whether pharmacy professionals would be able to access and interpret the up-to-date clinical information required to make informed medication related decisions.

*I’m on blood pressure *tablets* and he [GP] will say to me ‘Oh see the nurse and have an update with how your blood pressures going along...’ I don’t think the pharmacist would actually say that… I don’t think he would go … ‘When was the last time you had a test to see how your diabetes was?’… I just feel that a doctor would check all those things and especially knowing the patient and seeing the patient ongoing, all of the time, a doctor would notice that. I don’t know if a pharmacist would (Mrs L, aware)*

Some participants talked of secondary care as being less patient centred than primary care and welcomed the opportunity offered in general practice for shared decision making in relation to their medication use.

*I go into hospital … “We’re *going* to put you on what we think,” … by the time I get back, I go to the doctor [GP] and I’m saying, “Look, this one isn’t working.” “No, we’ll go back to what we know will work.” They see me all the time, hospitals don’t. (Mrs O, aware)*

Participants acknowledged that medication review via telephone avoided the need to go to the practice, however expressed concerns that efficacy of communication would be compromised, and information misheard or misunderstood. Participants strongly expressed a preference for face-to-face medication review in situations where changes to medicines were being proposed.

*I would have preferred face to face… I would like to have spoken to somebody directly … so that I didn’t have to shout... I felt very much like I was being processed (Mrs C, aware)*

*That’s when you actually must speak to the patient, … preferably face to face …. You don’t just alter something without going through the whys, wherefores and hows ... I wouldn’t be happy with that (Mr I, aware)*

### Output from the codesign phase

PPI advisers suggested using research findings to develop a patient information leaflet containing information about the purpose of medication review, what it involves and guidance on how to prepare for it. Advisers recommended that the leaflet explain that medication review may be led by a pharmacist who as a member of the practice team was a highly qualified expert in both medicines and the management of long-term conditions. To prompt patient reflection on their medication use, advisors recommended the use of example questions the patient may like to raise and that the reviewer may be expected to ask the patient during the review. Advisors felt strongly that a free-text space be included for patients to note medicines and concerns they wished to discuss with their reviewer. The resulting patient information leaflet is presented as Supplementary Material.

## Discussion

### Summary of main findings

Participants described a range of expectations and experiences of medication review, with some valuing medication review and others describing it as uneventful and unhelpful. Many of those that attached little value to medication review also described feeling uninformed and uncertain of the intended purpose. Participants who were receiving fewer medicines and held strong beliefs that their existing medicines posed little harm considered medication review to be more appropriate and beneficial for other people, particularly those with poor medication literacy and adherence. Participants also expressed preferences related to communication from the practice prior to medication review. Further information around the intended purpose and nature of medication review was desired by those who were less familiar with it. With respect to the mode of delivery of the review, face-to-face rather than telephone consultations were favoured explicitly where changes to prescribing were being discussed. Relationship continuity was generally regarded as salient to efficient and effective medication review. Whilst pharmacists were thought to have greater knowledge of medicines than GPs, several participants were unfamiliar with the role of pharmacists within general practice and expressed a lack of confidence in the clinical skills and knowledge of pharmacy professionals. Opinions related to the clinical capabilities of pharmacists were predominantly formed based upon patient experiences of discussing medicines use with pharmacists working in a community setting during a Medicines Use Review (a pharmacist-led review focused on how patients use their medicines, including how they should be taken, why they have been prescribed and identifying problems to feedback to the prescriber) [17] or when medicines were being dispensed, rather than being informed by direct experience from consulting a pharmacist working within a primary care setting.

### Strengths and limitations

Strengths of the current study include the focus on an in-depth understanding of patients’ perspectives and experiences of medication review, the recruitment of a diverse sample and the attainment of data saturation. The recruitment strategy sought to identify patients who had experienced a recent medication review with either a GP or a practice pharmacist in routine practice. Notably, only half of the participants were aware of or could recall having received a medication review. This, however, reflects the real-world practice of medication review where some reviews are being conducted remotely, without patient involvement or briefly, during routine consultations alongside discussion of other issues [12]. The sample also comprised some participants who did not have direct experience of a pharmacist-led or telephone review. Nonetheless all participants provided rich data on what they would like a review to look like in terms of organisation, delivery, and content and which reflect realities that are likely to resonate with the views and experiences of many patients across the UK. Our findings provide new perspectives to prompt critical reflection on clinical practice and policies for medication reviews. The study was conducted in practices in one geographical location and findings should be interpreted in light of this limitation. In addition, new ways of working have recently been implemented in Primary Care due to the COVID-19 pandemic, and the current study does not reflect any resulting changes in patients’ and healthcare professionals’ approaches to medication review.

### Comparison with existing literature

Findings are consistent with a focus group study with older patients who had attended a pharmacist-led clinical medication review in general practice which found that some people did not understand or were suspicious about the purpose of medication review [18]. However, in contrast to participants in the focus group study, our participants assumed that non-attendance at review would lead to their medicines being stopped and did not voice beliefs that the aim of review was to switch medicines to more cost-effective alternatives. Despite the patient profile, no participants in our study reported having a carer. Consonant with the studies by Petty et al. and Uhl et al. [18, 19] there was scepticism about the need for medication review for all patients with polypharmacy. This opinion was not linked to whether the participant had regular contact with primary care professionals [18], although was associated with patient autonomy as shown by others [17]. In addition, the current study found this view was explicitly expressed by participants receiving fewer medicines which they deemed to pose little harm.

The findings provide further evidence of the importance of relationship continuity to patients [20]. In the current study, a preference for relationship continuity by default valued GP-led above pharmacist-led medication review, as GPs are the primary prescribers and care coordinators in this setting. As shown in other studies, pharmacists were generally regarded as professionals with specialist expertise in medicines, but there was a lack of awareness of pharmacists’ clinical roles and capabilities [21–23]. The latest GP five-year contract framework commits to increasing the number of pharmacists working in primary care over the coming years [11]. Patient attitudes towards and acceptance of pharmacist-led medication review are therefore likely to change as these professionals become more integrated within general practice teams and take greater responsibility for medicines optimisation in patients with polypharmacy. The role of pharmacists in general practice has been investigated internationally [24, 25]. Studies conducted recently in England, Australia, Canada and New Zealand suggest that pharmacists’ scope of practice within primary care is developing and evolving towards a more clinically orientated role with telephone support for patients, medication review, medicines reconciliation following discharge/transfer of care and management of long-term condition as main roles [26–29].

Although there is a strong deprescribing movement, and greatly increased awareness of polypharmacy as a challenge for health services, many study participants described their review as a brief, box ticking exercise in which no changes to medicines were made. This suggests that medication reviews were primarily being offered as a means of ensuring the safe and effective ongoing use of long-term regular medications and were not necessarily focused on deprescribing. A key finding of our study is that, despite having a medicine review recorded in their GP records, many participants did not recall having a review. This finding is in keeping with a study by Duncan et al. in which GPs and pharmacists from the UK reported little or no patient involvement in medication review [12]. Lack of patient involvement in medication review is an important barrier to deprescribing (stopping or tapering down medications) and one possible explanation for increasing rates of polypharmacy [30].

### Implications for practice

Findings suggest that existing processes prior to medication review provide limited opportunity for patients to prepare for and engage in medication reviews. Furthermore, a lack of understanding of the intended purpose of medication review and what is involved appears to negatively impact patient experience. There is therefore likely to be considerable benefit in improving prior communication about the purpose and potential benefits of medication review. Moreover, patients in the current study expected their reviewer to focus on and address their medication related concerns. To further enhance the patient’s experience of medication review, reviewers should provide an opportunity for patients to ask questions and discuss specific problems and/or concerns.

Study findings also highlight that some patients are unfamiliar with the roles and responsibilities of pharmacists within general practice. Further work to raise patient awareness of the clinical responsibilities and skills of pharmacists may help facilitate integration of these professionals within general practice teams.

A patient information leaflet containing guidance for patients on what to expect and how to prepare for a medication review has been co-produced by the research team and PPI advisors. Provision of this resource prior to medication review may help to improve patient engagement and facilitate greater patient involvement in discussion and decision making around medicines use during medication review. The implementation and acceptability of the information leaflet to support patient preparation for medication review will be examined within a pragmatic process evaluation in a real-world setting.

In conclusion, the results of this research highlight important deficiencies in the way in which medication reviews are implemented in general practice. There is a need to address these problems to ensure maximum benefit is achieved by reviews and that they effectively address patients’ needs and concerns.

## Supplementary Information

**Additional file 1.**

## Data Availability

The data used to support the findings of this study are included within the article. Raw data analysed during the study are not publicly available due to confidentiality agreements but can be made available from the corresponding author in reasonable request.
