# Supplementary figures and images for: Patient perceptions and experiences of medication review: qualitative study in general practice
Source: BMC Prim Care. 2022 Nov 22;23:293. doi: 10.1186/s12875-022-01903-8 (PMC9682692; doi:10.1186/s12875-022-01903-8)

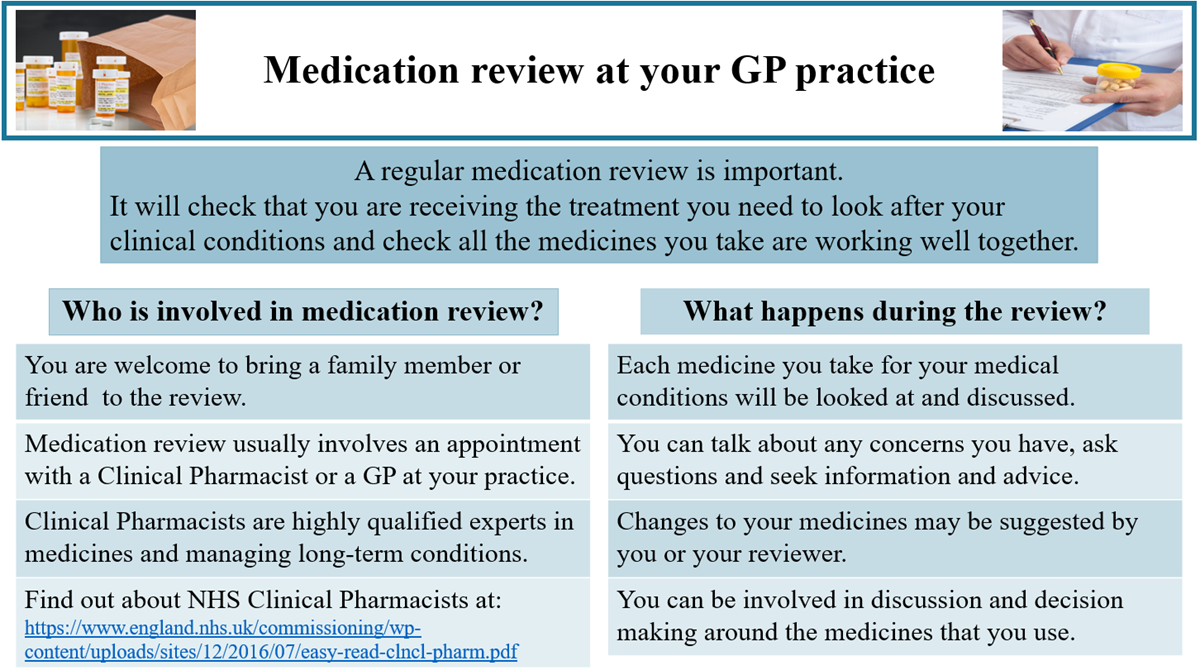


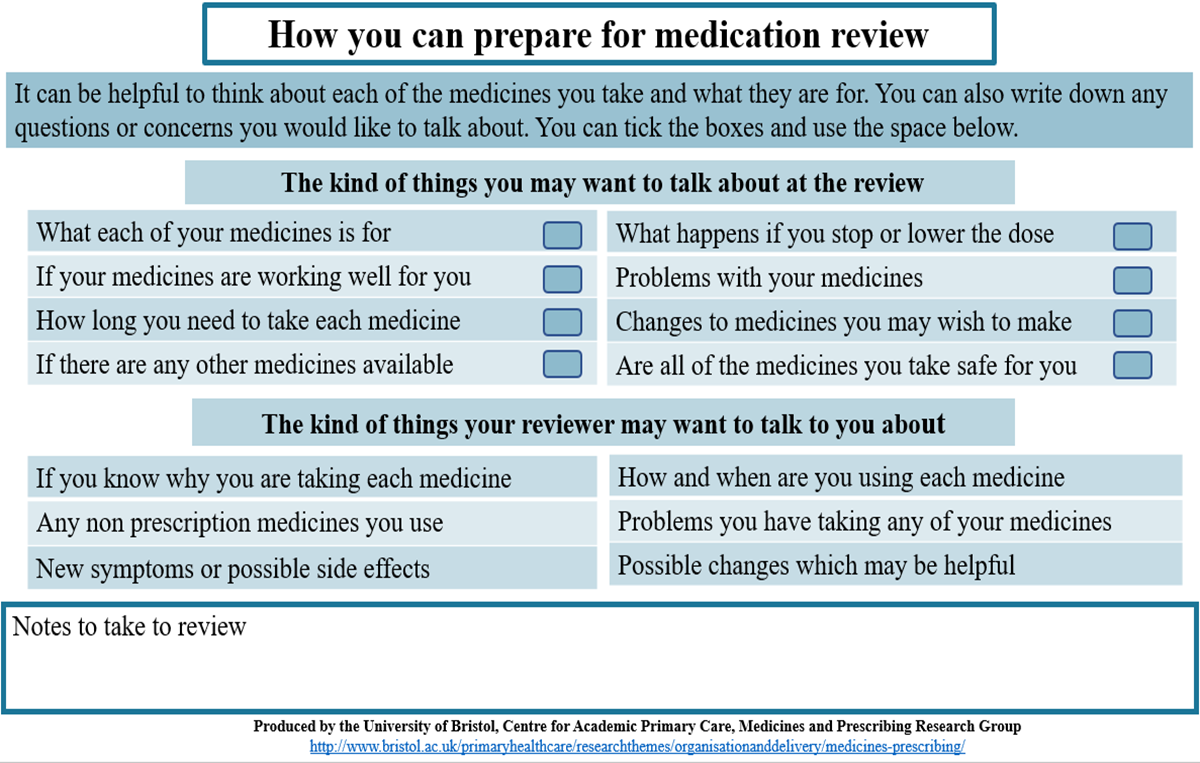

Supplement: Supplementary file 1 — Additional file 1. [file 12875_2022_1903_MOESM1_ESM.docx]
